# Higher adrenomedullin levels during implantation are associated with successful pregnancy

**DOI:** 10.2144/fsoa-2019-0147

**Published:** 2020-01-14

**Authors:** Pervin Karli, Fatma D Bildircin, Ayse Z Ozdemir, Bahattin Avci

**Affiliations:** 1 Department of Obstetrics & Gynecology, Amasya University Research Hospital, Amasya, Turkey; 2 Ondokuz Mayis University IVF Center, Ondokuz Mayis University Hospital, Samsun, Turkey; 3 Department of Biochemistry, Faculty of Medicine, Ondokuz Mayis University, Samsun, Turkey

**Keywords:** *in vitro* fertilization, adrenomedullin, embryo, embryo transfer, endometrium, implantation, infertility, placentation, pregnancy, serum

## Abstract

**Aim:**

To determine how the adrenomedullin (ADM) level in a woman’s serum on the day of embryo transfer affects pregnancy results.

**Materials & Methods:**

Women who had undergone frozen embryo transfer between July 2018 and February 2019 were prospectively included in the study. The relation between the level of ADM and pregnancy result was examined after taking a sample of serum from each patients on the same day as the transfer.

**Results:**

The results revealed that the ADM levels in patients who became pregnant were higher, but not to a statistically significant level.

**Conclusion:**

Adrenomedullin is an important molecule for human embryo implantation.

## Lay abstract

Implantation is the most important stage of *in vitro* fertilization. Despite a great many improvements in *in vitro* fertilization, the mechanism behind embryonic implantation remains a challenge. Adrenomedullin (ADM) is one of the molecules that has been researched to determine its role in implantation; building on this work, the present study aimed to show the effect of ADM on implantation. This study makes a crucial contribution to the literature, providing more information about how ADM affects the implantation process in humans.

Despite many improvements in the process of *in vitro* fertilization (IVF), almost half of all euploid embryos fail to implant in the endometrium [1]. Implantation is realized via a complex interaction between the embryo and the endometrium. Adrenomedullin (ADM) is one of molecules that have been researched as a possible means of boosting implantation success [2,3]. ADM is a protein that is present in the reproductive organs; it is generated by the female reproductive system [4]. Specifically, it is thought to play a role in implantation and placentation [5]. ADM suppresses uterine natural killer (NK) cells and ensures spiral artery remodeling in the uterus, facilitating the implantation and healthy placentation of the embryo [5]. One study carried out on animals revealed that ADM administered to the endometrium before embryonic transfer boosted implantation [6]. Another study demonstrated that the formation of pinopodes in the endometrium diminished; further, implantation was distorted when the researchers manufactured a defect of the ADM in animal subjects [7]. However, no study using human subjects has looked into the relationship between the level of ADM in the serum and implantation success. Thus, the present study attempted to investigate how the level of serum ADM on the day of the transfer affected pregnancy results.

## Patients & methods

Patients who had undergone frozen embryo transfer on Day 5 at Ondokuz Mayis University, Center of *In Vitro* Fertilization, between July 2018 and February 2019 were included in this prospective cohort study. The ethical committee of Ondokuz Mayıs University approved the study. All of the participants gave their written informed consent.

### Inclusion criteria

Patients aged 45 and below who had undergone embryo transfer for the first time were included in the study. Only patients that underwent frozen embryo transfer on Day 5 were included.

### Exclusion criteria

None of the included patients had any type of endocrine disease, such as diabetes or hypothyroidism. Fresh embryo transfers were not included in the study, as they were subject to hormonal fluctuations stemming from ovulation induction. Transfers other than those on Day 5 of the embryo were also excluded from the study for the purpose of forming a homogenous group. Patients with endometriosis, polycystic ovarian syndrome, who had undergone testicular sperm extraction, with myoma uteri, uterine anomalies and patients prone to difficult transfer process were excluded from the study. Finally, patients whose endometrium thickness was below 7 mm before the transfer were excluded from the study.

### Ovulation induction

The patients were examined on Day 2 or 3 of menstruation, and the gonadotropin follicle-stimulating hormone (FSH) (Gonal-F; Serono, Germany) implementation was applied. The gonadotropin-releasing hormone antagonist 0.25 mg cetrorelix acetate (Cetrotide: 0.25 mg; Serono, Germany) was added when the diameter of the follicle reached 12 mm. Recombinant human chorionic gonadotropin (hCG) (Ovitrelle: 250 μg; Serono, Germany) was administered once two follicles reached 17 mm in diameter. Oocyte pickup was performed 36 h later following hCG administration; then, intracytoplasmic sperm injection was performed. The embryos were frozen on Day 5.

At the center, preparations for all frozen transfers regarding the endometrium are made using hormone replacement therapy. In all the patients in the present study, endometrium preparation was initiated using estrogen (Estrofem: 2 mg; Novo Nordisk, Denmark) on menstrual cycle Days 2–3 following transvaginal ultrasonography. The endometrium preparation protocol began with 4 mg/day of estrogen on Days 1–4, 6 mg/day on Days 5–8 and 8 mg/day from Day 9 onward. A second transvaginal ultrasonography was performed following 10 days of estrogen treatment. Embryo transfer was scheduled in cases where the endometrial thickness was at least 7 mm. Progesterone was administered intramuscularly (Progestan 50 mg; Kocak, Turkey) at a dose of 100 mg for five complete days prior to embryo transfer.

The resulting embryos were then graded for quality according to their morphological characteristics; they were assigned a score between 1 (best) and 5 (worst) in terms of the regularity of the blastomers, the percentage of anucleate fragments and all their dysmorphic characteristics. Grade 1: 0% anucleate fragments, regular blastomers and no apparent morphologic abnormalities; grade 2: less than 10% anucleate fragments, regularity of blastomers and no apparent morphologic abnormalities; grade 3: 10–50% anucleate fragments, irregularity of blastomers and no apparent morphologic abnormalities; grade 4: ≥50% anucleate fragmentation, irregularity of blastomers and apparent morphologic abnormalities. Grade 1–3 embryos were transferred. All the transfers were performed without anesthesia using ultrasonography by the same reproductive endocrinologist. Progesterone was given intramuscularly (Progestan: 50 mg; Kocak), and estrogen (Estrofem: 2 mg; Novo Nordisk) was given orally as luteal support until 12 weeks of pregnancy.

### Taking & examining serum samples

Serum samples were taken prior to embryo transfer from all patients on the day of the transfer and centrifuged for 10 min at 3000× *g* (Shimadzu UV160A, SNo: 28006648, Kyoto, Japan). The samples were kept at -80°C until the day of the study. The samples were at room temperature on the day of the study.

### Determination of human ADM concentrations

ADM concentrations in human serum were measured through the commercially available Human ADM Enzyme Linked Immunosorbent Assay (ELISA) kit (Cat No. 201-12-1025; Sun-Red Bio Company, Shanghai, China) via double-sandwich and enzyme immunosorbent assay methods. The measurements were done at Ondokuz Mayis University Faculty of Medicine, Medical Biochemistry Department Research Laboratory. All the solutions for the study were freshly prepared and placed at room temperature (25°C) before use.

Five standards (S1: 50 ng/l, S2: 100 ng/l, S3: 200 ng/l, S4: 400 ng/l and S5: 800 ng/l) were prepared via serial dilution using the human ADM standard. An ELISA plate was used for the blank wells, standard wells and sample wells. Nothing was added to the blank wells other than Chromogen A, Chromogen B and a stop solution. The same procedure was applied to the standards as the one applied to the samples. A 50-μl standard (S1–S5) was pipetted into each well; then, 40 + 10 μl ADM antibody was pipetted from each sample. After that, 50 μl Streptavidin-Horse Radish Peroxidase was added to the standards and samples, and they were left to incubate for 60 min at 37°C. In the subsequent stage of incubation, the plate was washed five-times with 350-μl washing solution with the aid of an automatic washer. Then, 50-μl Chromogen A and 50-μl Chromogen B were added to all wells, and they were left to incubate for 10 min at 37°C. After this, 50 μl of a stop solution was pipetted into the wells, ceasing the reaction. At the end of the study, absorbances at the wavelength of 450 nm were measured using the TECAN microplate reader.

Sample human ADM concentrations were calculated in accordance with the standard line produced using the values of the standards; the concentrations obtained were expressed as ng/l. Kit sensitivity was specified as 5.118 ng/l, and the assay range was expressed as 7–1500 ng/l. The samples with high concentrations were validated after being examined twice.

### Outcome measure

The diagnosis of pregnancy was made via a positive hCG test, which was taken on Day 14. The diagnosis of an ectopic pregnancy was made through ultrasonography. Abortus was defined as the loss of the pregnancy within the first 20 weeks of pregnancy.

### Statistical analysis

In calculating the sample amplitude of this study, the power (power of the test) was determined to be 0.80; the type 1 error was determined to be 0.05 for each variable. The descriptive statistics for the continuous (quantitative) variables are expressed as medians, averages, standard deviations, and minimum and maximum values. Whether the continuous variables in the study were distributed normally or not were examined using the Kolmogorov–Smirnov test (N >50). Nonparametric tests were conducted because the variables were not distributed in a normal fashion. The Mann–Whitney U test was employed to compare the measurements for each pregnancy group. Receiver-operating characteristic analysis was employed to determine the cutoff values of the parameters of FSH and ADM in the pregnancy groups from the standpoint of their values of sensitivity and specificity. Spearman’s correlation coefficients were calculated for the purpose of determining the relationship among variables. A Chi-square test was used to examine the relationship between the categorical variables. Statistical significance level was accepted as %5 in calculations. Finally, the statistical package for the social sciences (SPSS; IBM SPSS for Windows, Ver. 24) was employed to conduct the calculations.

## Results

The demographic data are shown in Tables 1 and 2. No difference was detected in the pregnant and not pregnant groups in terms of age, thickness of the endometrium, number of oocytes collected, levels of estradiol (E2) and levels of FSH (p > 0,05). The embryo grade and the number of embryos transferred exhibited no statistically significant change in either the pregnant or the not pregnant groups (p > 0.05; Tables 1 & 2).

**Table 1.** Demographic data 1.

Demographic	Not pregnant					Pregnant					p-value
	Median	Mean	SD	Min	Max	Median	Mean	SD	Min	Max	
Age	30	31.27	4.79	24	40	31	31.46	5.03	21	40	0.73
Thickness of the endometrium	11	10.6	1.53	6	17	11	10.76	1.29	7	13	0.29
Number of collected oocytes	9	9.52	4.48	2	22	8,5	9.26	4.29	3	24	0.677
FSH	8	10.91	7.26	3	35	8	9.39	4.92	4	25	0.459
Estradiol on oocyte pickup day	2025	2002.34	905.19	450	4600	1890	1964.63	899.05	540	5150	0.729

FSH: Follicle-stimulating hormone; SD: Standard deviation.

**Table 2.** Demographic data 2.

Demographic data	Not pregnant		Pregnant		p-value^†^
	N	%	N	%	
**Reason for infertility**					
Unexplained infertility	56	59.6	39	72.2	0.179
Male factor	24	25.5	7	13.0	
Diminished ovarian reserve	14	14.9	8	14.8	
**Grade**					
1	83	88.3	44	81.5	0.118
2	10	10.6	6	11.1	
3	1	1.1	4	7.4	
**Number of embryos transferred**					
1	5	5.3	1	1.9	0.303
2	89	94.7	53	98.1	

† Significance levels of Χ^2^ test.

Although the ADM average of the pregnant group was found to be higher (641.38) than that of the not pregnant group, the difference was not statistically significant (Table 3).

**Table 3.** Adrenomedullin levels in the pregnant and not pregnant groups.

	Not pregnant					Pregnant					p-value^†^
	Median	Mean	SD	Min	Max	Median	Mean	SD	Min	Max	
ADM (ng/l)	639.12	636.25	78.49	387.35	801.36	641.38	635.82	67.19	519.54	800.14	0.692

† Significance levels of Χ^2^ test.

ADM: Adrenomedullin; SD: Standard deviation.

The correlation coefficients among the continuous variables for the patients are given in Table 4. The correlation coefficients belonging to the measurements with significant relationships are marked with an asterisk. When examined in accordance with this, a statistically significant inverse relationship (at the level of 70.7%) was observed between FSH and the number of oocytes collected (p < 0.05). In a similar way, a significant relationship was also observed between the number of oocytes collected through E2 and E2 and FSH. In contrast, there was no significant relationship between ADM and other variables (p > 0.05; Table 4).

**Table 4.** Correlation coefficients among continuous variables.

Variable	ADM	Age	Endometrium	Number of oocytes	FSH
Age (r)	0.019				
Endometrial thickness (r)	0.003	-0.052			
Number of collected oocytes (r)	0.034	0.057	0.059		
FSH (r)	0.030	-0.084	-0.046	-0.707^†^	
Estradiol on the day of oocyte pickup (r)	0.026	0087	0.052	0.972^†^	-0.671^†^

† p < 0.01.

ADM: Adrenomedullin; FSH: Follicle-stimulating hormone; r: Spearman’s rho nonparametric correlations coefficients.

Table 5 illustrates the comparison results of the ADM measurements in the categorical data. The level of ADM had no significant effect on ectopic pregnancy and abortus (Table 5).

**Table 5.** Comparison results of adrenomedullin levels in the categorical data.

State	ADM (ng/l)					p-value^†^
	Median	Mean	SD	Min	Max	
Not pregnant	639.12	636.25	78.49	387.35	801.36	0.692
Pregnant	641.38	635.82	67.19	519.54	800.14	
Nonectopic	641.38	636.14	74.99	387.35	801.36	0.913
Ectopic pregnant	633.40	633.40	.	633.40	633.40	
No abortus	640.53	636.53	74.38	387.35	801.36	0.699
Abortus	647.43	629.34	85.51	519.54	748.97	

† Significance levels of the Mann–Whitney U test.

ADM: Adrenomedullin; SD: Standard deviation.

**Figure 1.**
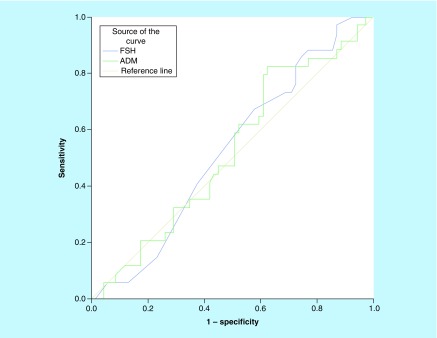
Image of adrenomedullin sensitivity (receiver-operating characteristic curve). ADM: Adrenomedullin; FSH: Follicle-stimulating hormone.

The areas under the curve and the cutoff values of the FSH and ADM variables are expressed as the receiver-operating characteristic curve (Figure 1).

The cutoff values demonstrating the best (optimum) sensitivity and specificity values of these variables are given in the table. These values serve as distinguishing values for the pregnant and not pregnant groups. In accordance with this, the sensitivity and specificity values were calculated, respectively (FSH: sensitivity 67.6% and specificity 42.0%; ADM: sensitivity 58.8% and specificity 49.3%; Table 6).

**Table 6.** Area under the curve and cutoff values in accordance with state of pregnancy.

Test result variables	Area under the curve	Std error	p-value	Cutoff	Sensitivity	Specificity
FSH	0.527	0.058		8.50	0.676	0.420
ADM	0.524	0.059		646.82	0.588	0.493

The test result variable(s) FSH and ADM have at least one link between the positive actual state group and the negative actual state group.

ADM: Adrenomedullin; FSH: Follicle-stimulating hormone.

## Discussion

ADM is an angiogenic, anti-inflammatory and vasodilatory protein that was first detected in pheochromocytoma [8]. It is a protein that is effective in cardiac, lymphatic and vascular systems as well as in tumors [9]. It is in calcitonin gene-related peptide family and functions in tissues by binding to G receptors. Hypoxy, estrogen and progesterone boost ADM synthesis in reproductive tissues and in the placenta [10]. Despite that the level of serum ADM increases in the body during pneumonia, sepsis and cardiac disease, the highest increase is observed during pregnancy [11].

ADM is secreted by the macrophage inside the follicles and granulosa cells [12]. It has been found in higher levels in follicular liquid than in plasma. ADM levels increase as the estradiol level in a follicle increases. Also, the amount of ADM in a follicle indicates the oocyte and embryonic quality [13]. ADM boosts the FSH response of a follicle and regulates the development of follicles and luteinization. It is also effective in angiogenesis. Finally, it plays a role in the secretion of gonadotropin from the pituitary gland.

Related to implantation, Matson *et al.* [6] found that when they administered ADM to the endometrium in mice, it boosted water transport and the formation of pinopodes in epithelia. It was beneficial for implantation in how it boosted connexin 43 and gap junction in the stroma. Low levels of ADM spoil the immunological harmony between the fetus and the mother, causing abnormal placentation and complications [5]. One study observed that levels of mid-regional pro-ADM were low in patients with serious preeclampsia [14].

The present study researched serum ADM levels on the day of embryo transfer for the purpose of observing its effect on implantation. Despite the findings not being statistically significant, the serum ADM levels were observed to be higher in pregnant patients than in not pregnant patients.

As is well known, implantation is the most important step of IVF therapy. However, the specific interactions between the embryo and the endometrium are not fully understood. There are a lot of mediators that may be effective in implantation. ADM is one of these, with animal studies indicating that it plays an important role in implantation. This is the first study, to the authors’ knowledge that reports on the effect of ADM on implantation in humans.

One of the limitations of the study is that the levels of ADM at the beginning of the IVF cycles are not mentioned. The study also did not indicate that the embryos were euploid embryos. The perinatal results cannot be given because long-term monitoring of the patients was not conducted. Finally, the endometrial levels of ADM were not mentioned, which would show the importance of the local effect.

## Conclusion & future perspective

Embryo implantation is a complex process and represents a speed constraint in IVF. Despite the enormous improvements in IVF, the implantation step remains a mystery. Animal studies have confirmed that ADM enhances embryo implantation. The present study is the first study looking into the relationship between ADM and implantation in humans and shows that ADM may enhance the pregnancy rates associated with IVF. However, more prospective, large-scale studies are needed to explore these results further, the results of which may shed light on which molecules may be used to enhance implantation and pregnancy rates.

**Summary points**

- Adrenomedullin (ADM) is a protein that has been shown to enhance embryo implantation in animal studies.
- There has been no study about the effect of ADM on human embryo implantation.
- In our pregnant group, serum ADM levels were higher despite not being statistically significant.
- We need larger prospective studies to further examine the effect of ADM on implantation.
